# An Autochthonous Susceptible *Candida auris* Clade I Otomycosis Case in Iran

**DOI:** 10.3390/jof9111101

**Published:** 2023-11-11

**Authors:** Bahram Ahmadi, Behrouz Naeimi, Mohammad Javad Ahmadipour, Hamid Morovati, Theun de Groot, Bram Spruijtenburg, Hamid Badali, Jacques F. Meis

**Affiliations:** 1 Department of Medical Laboratory Sciences, Faculty of Paramedical, Bushehr University of Medical Sciences, Bushehr 75187-59577, Iran; bahram_sound2005@yahoo.com (B.A.); b.naeimi1350@gmail.com (B.N.); 2 Department of Ear, Nose & Throat (ENT), Dey Clinic, Bushehr 75187-59577, Iran; drahmadipour@yahoo.com; 3 Department of Parasitology and Mycology, School of Medicine, Shiraz University of Medical Sciences, Shiraz 71348-14336, Iran; morovatihamid1989@gmail.com; 4 Department of Medical Microbiology and Infectious Diseases, Canisius-Wilhelmina Hospital, 6532 SZ Nijmegen, The Netherlands; t.degroot@cwz.nl (T.d.G.); bram3012@gmail.com (B.S.); 5 Center of Expertise for Mycology, Radboud University Medical Center/Canisius-Wilhelmina Hospital, 6532 SZ Nijmegen, The Netherlands; 6 Department of Molecular Microbiology & Immunology, South Texas Center for Emerging Infectious Diseases, The University of Texas, San Antonio, TX 78249, USA; 7 Institute of Translational Research, Cologne Excellence Cluster on Cellular Stress Responses in Aging-Associated Diseases (CECAD), Excellence Center for Medical Mycology (ECMM), University of Cologne, 50923 Cologne, Germany

**Keywords:** *Candida auris*, clade I, otomycosis, Iran, whole genome sequencing, genotyping

## Abstract

*Candida auris* is a newly emerging multidrug-resistant fungal pathogen considered to be a serious global health threat. Due to diagnostic challenges, there is no precise estimate for the prevalence rate of this pathogen in Iran. Since 2019, only six culture-proven *C. auris* cases have been reported from Iran, of which, five belonged to clade V and one to clade I. Herein, we report a case of otomycosis due to *C. auris* from 2017 in a 78-year-old man with diabetes mellitus type II without an epidemiological link to other cases or travel history. Short tandem repeat genotyping and whole genome sequencing (WGS) analysis revealed that this isolate belonged to clade I of *C. auris* (South Asian Clade). The WGS single nucleotide polymorphism calling demonstrated that the *C. auris* isolate from 2017 is not related to a previously reported clade I isolate from Iran. The presence of this retrospectively recognized clade I isolate also suggests an early introduction from other regions or an autochthonous presence. Although the majority of reported *C. auris* isolates worldwide are resistant to fluconazole and, to a lesser extent, to echinocandins and amphotericin B, the reported clade I isolate from Iran was susceptible to all antifungal drugs.

## 1. Introduction

*Candida auris* is a newly described emerging multidrug-resistant yeast which is regarded as a serious global health threat [1,2,3,4]. This pathogen was initially described in a Japanese patient with otitis externa in 2009 [5], followed by outbreaks in India in 2012–2014 [6] and a global emergence from 2015 onwards [2,3,7,8]. The recent studies of *C. auris* have pointed to environmental and/or animal niches [9,10,11]. COVID-19 infection, diabetes mellitus (DM), immunosuppression, broad-spectrum antimicrobial use, and corticosteroids are among the main risk factors for *C. auris* infections [3,11,12]. Compared to other pathogenic fungi, *C. auris* has unique abilities such as long-term persistence on inanimate surfaces and protracted cross-transmission between patients [13]. Furthermore, a significant percentage of *C. auris* isolates have been shown to be resistant to or have reduced susceptibility to registered antifungal drugs [14]. Based on genomic analyses, *C. auris* isolates have been allocated to five geographical clades (I to V): the South Asian, East Asian, African, South American, and Iranian clade, respectively [8,15], while an additional clade (VI), Indomalaya, was recently reported in Singapore [16]. These distinct *C. auris* clades evolved seemingly simultaneously at independent geographical locations and display both genetic and phenotypic diversity [8]. While clades I, III, and IV are responsible for the ongoing outbreaks of invasive and multidrug-resistant infections, clades II and V consist primarily of cases of ear infections, are often susceptible to antifungal drugs, and have not been associated with outbreaks [15,17]. Here, we describe a case of *C. auris* otomycosis of a patient in Bushehr, Iran, compare this with earlier reported cases, and performed susceptibility testing on the isolate.

## 2. Case Presentation

In April 2017, a 78-year-old man with discharge from and itching of the external ear canal was admitted to an ear, nose, and throat specialist in Bushehr, a coastal city at the Persian Gulf, Iran. He had diabetes mellitus type II for more than 10 years as the only predisposing factor. With the clinical suspicion of a fungal infection, the ear discharge was submitted for investigation. Round and oval cells were observed with microscopic examination with 10% potassium hydroxide (KOH). The specimen was cultured on Sabouraud’s dextrose agar supplemented with chloramphenicol (SDA, Merck, Darmstadt, Germany), and incubated at 35 °C for 48 h. White-to-cream, smooth, yeast-like colonies were germ tube-negative and thus identified as non-*albicans Candida* species. The patient was managed with topical empirical therapy with betamethasone 1% for the itching and inflammation of the external ear canal, as well as hydrogen peroxide 1% and ciprofloxacin 0.3%. The patient completely improved after one week with this regimen and did not receive specific antifungal therapy.

## 3. Materials and Methods

Retrospective molecular identification was performed 5 years later with ITS rDNA sequencing [18,19] and matrix-assisted laser desorption/ionization time-of-flight mass spectrometry (MALDI-TOF; Bruker, Bremen, Germany) identifying the yeast as *C. auris.* For DNA isolation, the isolate was resuspended in 700 µL MagNA Pure bacteria lysis buffer and MagNA Lyser green beads and lysed with the MagNA Lyser system (all Roche Diagnostics GmbH, Mannheim, Germany). Subsequent DNA extraction and purification was performed with the MagNA Pure 96 instrument and the MagNA Pure DNA and Viral NA Small Volume kit (Roche Diagnostics) [20,21]. The ITS rDNA sequence of the isolate (MRL32) was deposited in GenBank under accession number OQ740733. In vitro antifungal susceptibility testing was performed by broth microdilution according to the Clinical and Laboratory Standards Institute (CLSI) standard M27-S4 [22]. In addition, short tandem repeat (STR) genotyping was performed as previously described [20]. For whole-genome sequencing (WGS), genomic libraries were prepared and sequenced with the Illumina NovaSeq 6000 platform (Illumina, San Diego, CA, USA) with 2- by 150-bp paired-end read mode at Eurofins Genomics (Ebersberg, Germany). Read data were extracted from the SRA database (Supplementary Table S1). Reads were aligned and variant calling was performed as previously described [23]. All raw read data generated in this study were deposited under BioProject accession number PRJNA1002104.

## 4. Results

**Figure 1:**
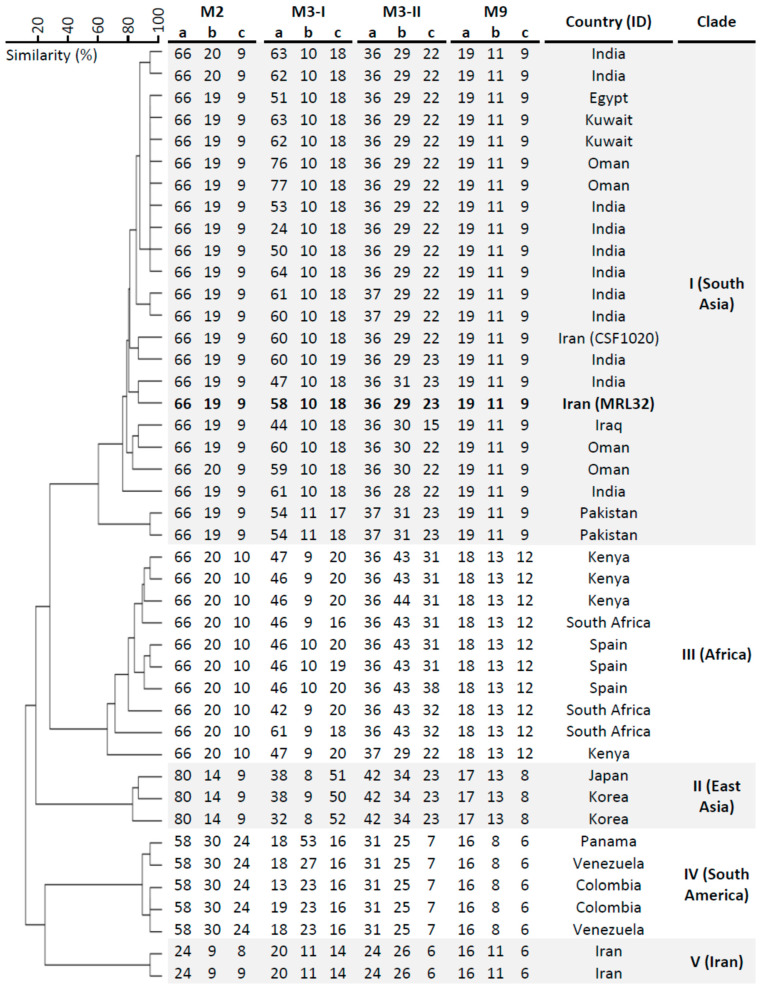
Short tandem repeat (STR) typing of *Candida auris* isolates including the current isolate. The UPGMA dendrogram was generated with BioNumerics v7.6, branch lengths indicate similarity, and the currently reported isolate is indicated in bold.

The STR dendrogram with the representatives of all known *C. auris* clades showed that MRL32 was phylogenetically distinct from other Iranian *C. auris* isolates belonging to the local clade V, clustered with the South Asian clade (I), but had a different genotype than the previously reported Iranian clade I isolate (CSF1020) (Figure 1).

**Figure 2:**
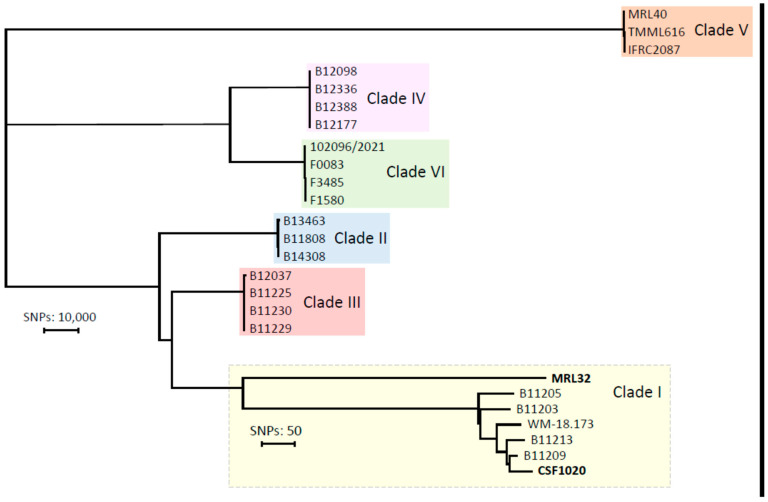
Phylogenetic tree based on single nucleotide polymorphisms (SNPs) of 29 *Candida auris* isolates including all reported clades. Numbers below the branch indicate the number of SNPs. The tree was generated with MEGA11 using the neighbor-joining tree method.

To determine the genetic distance between both Iranian clade I isolates, WGS single nucleotide polymorphism (SNP) calling was performed on both isolates and compared with other clades (Supplementary Table S1). The WGS SNP analysis confirmed that both Iranian isolates belonged to clade I and demonstrated that they were separated by 866 SNPs (Figure 2).

The minimum inhibitory concentrations of MRL32 were as follows: amphotericin B (1 μg/mL), fluconazole (8 μg/mL), itraconazole (0.5 μg/mL), posaconazole (0.25 μg/mL), voriconazole (0.125 μg/mL), isavuconazole (0.125 μg/mL), miconazole (4 μg/mL), caspofungin (0.25 μg/mL), anidulafungin (0.063 μg/mL), and micafungin (0.125 μg/mL). While the previously reported clade I fluconazole-resistant isolate (CSF1020) from Iran grouped with other fluconazole-resistant isolates from India, the fluconazole-susceptible isolate MRL32 did not group with any fluconazole-resistant isolates. In agreement with the phenotypic susceptibility testing, the visual alignment inspection with JBrowse of the resistance associated genes ERG11, TAC1b, and FKS1 showed the absence of unique mutations in MRL32 when compared to the resistant isolates. The previously reported fluconazole-resistant isolate from Iran was found to harbor the ERG11Y132F substitution.

The patient characteristics and diagnostic details of the present case and previously published Iranian *Candida auris* cases are depicted in Table 1.

## 5. Discussion

*C. auris* is an emerging multidrug-resistant pathogen becoming a global threat due to its potential for nosocomial spreading [28] during both the pre-COVID and COVID-19 era [11,29]. A systematic review and meta-analysis showed that the prevalence of *C. auris* among COVID-19 patients having DM was 52.9% [12] and that the pooled prevalence of *C. auris* infection among COVID-19 patients was 5.7%. DM is among the main predisposing factors of *C. auris* infection. Here, we report a retrospectively detected case from 2017 in a 78-year-old man with otitis externa caused by a susceptible *C. auris*. Up until now, the prevalence of *C. auris* in Iran was low [15,24,25,26,27]. The main reason for this low prevalence rate may be the inability to detect the pathogen and the lack of standard diagnostic methods. DNA sequencing is considered the gold standard method for identifying *C. auris.* However, diagnostic allele-specific PCR, STR typing, or whole-genome sequencing (WGS) are necessary for categorizing the geographical clades [3,20,30]. We show that the first Iranian *C. auris* from 2017 was actually from clade I. Retrospective studies from France also identified an early clade I introduction in 2007 [31]. The reported Iranian *C. auris* isolates from clade V had a similar susceptibility pattern as this clade I isolate from 2017 [15,24,25,26,27].

Our case is the sixth reported case of *C. auris* in Iran. The geographical distribution of *C. auris* cases in Iran is as follows: two cases from Babol in the north of Iran [15,24], one case from Isfahan in the center of Iran [26], one case from Shiraz (South of Iran) [27], one case from Tehran [25], and the present case from a Persian Gulf coastal city in the southwest of Iran, Bushehr. Most isolates belonged to the unique Iranian clade V, except the case from Tehran and the present one, which were both allocated to clade I. The clade V isolates differed by less than 100 single nucleotide polymorphisms (SNPs) intra-clade and by more than 200,000 SNPs between the known clades [15]. The antifungal susceptibility testing showed that two isolates (from Babol and Isfahan) were resistant to fluconazole. The in silico analysis of WGS reads indicated that the mechanisms of the underlying drug resistance in these two isolates was due to mutations in the TAC1b and ERG11 genes. The first reported South Asian (Clade I) *C. auris* isolate from Iran in 2021 was from an invasive infection linked to travel to the Indian subcontinent and was fluconazole-resistant [25]. Genetic analysis demonstrated the frequently reported ERG11Y132F substitution which is known to induce resistance [32].

Our patient is the oldest and the second male case of *C. auris* in Iran. Otitis externa was the most common presentation of *C. auris* (three out of six) in Iran. The case from Shiraz had epidermal dysplasia, while the Tehran case involved meningitis with *C. auris* in a 30-month-old male infant [25]. Earache, itching, discharge from the ear canal, and inflammation are the most common clinical signs and symptoms reported in Iranian *C. auris* cases. Among the patients with clade V isolates, none traveled abroad until the diagnosis of the infection, while the 30-month-old male infant from Tehran with clade I was hospitalized in Pakistan (Table 1).

Distinct *C. auris* clades evolved seemingly simultaneously at independent geographical locations and display both genetic and phenotypic diversity [8]. The emergence and persistence of four genomic *C. auris* clades in a single country without epidemiological links have been described in the USA [33], Canada [34], and Algeria [35], while the UK [36] and South Africa [37] reported three circulating clades. Many other countries, including Iran, have reported the presence of two clades.

To investigate the phylogenetic relationship between this isolate and the previously reported Iranian clade I isolate, WGS SNP analysis was performed on both. Due to substantial SNP differences (>800) between the isolates, it can be concluded that the introduction of the second clade I isolate was independent from the first isolate. The first isolate displayed low SNP differences when compared to other clinical-fluconazole resistant isolates, as is frequently observed for *C. auris* [21], and also correlated with previous hospitalization in Pakistan. Importantly, the 2017 clade I isolate from Iran did not group with the fluconazole-resistant isolates. In agreement, the fluconazole MIC was found to be wildtype, in contrast to most isolates from clade I that are fluconazole-resistant [38]. As there was no travel history of this patient from Bushehr, the mode of the introduction of this yeast in Iran remains to be determined. A study of an autochthonous clade I isolate in a Latin American country, where clade IV is most commonly encountered, also showed a large SNP difference and fluconazole susceptibility compared to the original clade I from the Indian subcontinent [39].

To conclude, misdiagnosis and the application of conventional diagnostic methods lead to a lack of data about *C. auris* cases in Iran. We found a second clade I isolate in Iran, which was not related to the previous clade I isolate. The addition of this and earlier reports to the literature may motivate researchers to focus on the detection of this pathogen.

## Figures and Tables

**Table 1:** Patient characteristics and diagnostic details of Iranian *Candida auris* cases.

		First Isolate	Second Isolate	Third Isolate	Fourth Isolate	Fifth Isolate	Sixth Isolate
[Original reference] year		[15] 2018	[24] 2021	[25] 2021	[26] 2021	[27] 2022	Current study
Geographical clade		V	V	I	V	V	I
Demographic Data	Age (year)	14	40	30 months	33	Teenager	78
	Sex	Female	Female	Male	Female	Female	Male
	Underlying diseases	Otitis	Bilateral otalgia	Meningitis	Otitis	Epidermal dysplasia (TB63 Genetic variant)	Diabetes mellitus
	Origin City and Zone	Babol; North of Iran	Babol; North of Iran	Isfahan; Center of Iran	Isfahan; Center of Iran	Shiraz; South of Iran	Bushehr; South-west of Iran
Site of Isolation		Ear discharge	Ear discharge	LP (CSF)	Ear discharge	- Ear discharge; - Skin scrapings	Ear discharge
Clinical Signs and Symptoms		- Right-sided earache; - Severe itching; - Hearing loss; - White-to-creamy discharge from the ear canal; - Inflammation; - Redness and tympanic membrane perforation in right ear	- Itching of external ear canal; - Creamy discharge of ear canal; - Inflammation (redness and swelling)	- Vomiting; - Loss of consciousness; - Frequent seizures	- Right-sided earache; - Severe itching; - Hearing loss; - White-to-creamy discharge from the ear canal; - Pruritus; - Otalgia; - Tinnitus; - Otorrhea; - Inflammation; - Redness in her right ear	- Severe itching; - Ear discharge	- Itching; - Ear discharge
Predisposing Factor		- No trauma or predisposing factor; -No travel history	- Repeated ear manipulation; - Ear eczema; - No travel history	- Previous hospitalization in Pakistan	- No predisposing factor; - No travel history	- Ichthyosis; - No travel history	- Diabetes mellitus type 2; - No travel history
Identification Methods	Mycological	- KOH smear; - Culture on SDA (white); - CHROM agar Candida (pink) - MALDI-TOF MS	- KOH smear; - Culture on SDA (white); - CHROM agar Candida (pale pink to dark purple)	- KOH smear; - Culture on SDA (white to creamy; yeast); - MALDI-TOF MS	- KOH and Giemsa smear (hyphae and yeasts); - Culture on SDA MALDI-TOF MS (filamentous fungi; *Aspergillus* sp.) (white to creamy; yeast); - CHROM agar Candida (pink)	- KOH smear; - Culture on SDA; - CHROM agar Candida; - MALDI-TOF MS	- KOH smear (round and oval cells); - Culture on SDA (white to cream); - CHROM agar Candida (pale pink to dark purple)
	Molecular	WGS	- PCR of *ITS* gene region WGS	- Multiplex PCR; - PCR of *ITS* gene region	- PCR of *beta tubulin* gene region; - PCR of *ITS* gene region; - STR genotyping WGS	- STR genotyping WGS	- Multiplex PCR of *ITS* gene region STR genotyping; -WGS
	Reference Sequencing	*ITS* rDNA [MK123931.1]	*ITS* rDNA [MW019910.1]	*ITS* rDNA MZ853742.1	*- Beta tubulin gene*; - *ITS* rDNA MZ389242.1	ND	*ITS* rDNA OQ740733
Similarity with the Standard/ex-type Isolate		99.5% with EU884189.1	100% with MH427523.1	100% with MT912579.1	- 99.5% with MT974681.1	ND	100% with OK625328.1
Antifungal Susceptibility Profile (μg/mL)		- Fluconazole: 16; - Itraconazole: 0.063 - Voriconazole: 0.125; - Micafungin: 0.031; - Amphotericin B: 0.5; - Anidulafungin: 0.016; - Posaconazole: 0.016; - Isavuconazole: 0.063	- Fluconazole: ≥32; - Itraconazole: 0.016 - Voriconazole: 1; - Micafungin: 0.032; - Amphotericin B: 0.25; - Anidulafungin: 0.016; - Posaconazole: 0.016; - Isavuconazole: 0.016; - Ravuconazole: 0.5; - Ketoconazole: 2; - Clotrimazole: 2 - Nystatin: 2	- Fluconazole: >64; Amphotericin B: 1; - Itraconazole: 0.125; - Voriconazole: 0.25; - Clotrimazole: 0.015; - Nystatin: 8; - Caspofungin: 0.5; - Anidulafungin: 1; - 5-flucytosine: 0.125	- Fluconazole: >64; - Itraconazole: 0.5; - Voriconazole: 1; - Micafungin: 0.015; - Caspofungin: 0.015; - Amphotericin B: 1; - Anidulafungin: 0.125; - Terbinafine: >16; - 5-flucytosine: 0.5	- Fluconazole: 1; - Itraconazole: 0.016; - Voriconazole: 0.016; - Micafungin: 0.031; - Amphotericin B: 0.25; - Anidulafungin: 0.63; - Posaconazole: 0.016; - Isavuconazole: 0.016	- Fluconazole: 8; - Itraconazole: 0.5; - Caspofungin: 1; - Amphotericin B: 1; - Miconazole: 4
Therapeutic Strategies		- Empirical antibacterial therapy: Cefixime (400 mg/d); Topical gentamicin - Antifungal therapy: Topical nystatin (100,000 Units/g); Terbinafine (250 mg/d)	- Empirical antibacterial therapy: Ciprofloxacin; Gentamicin - Inflammation therapy: Betamethasone - Antifungal therapy: Topical clotrimazole; Topical miconazole	- Empirical antibacterial therapy: Meropenem; Vancomycin - antiepileptic drugs: Phenobarbital; Phenytoin; Levetiracetam Inflammation therapy: IFN-γ - Antifungal therapy: Fluconazole (100 mg/kg/d); IV Liposomal amphotericin B (3 mg/kg/day; i.v); Oral flucytosine (250 mg/kg/d)	- Empirical antibacterial therapy: Myxacort; Bactimide - Surgery - Inflammation therapy: Hydrocortisone-acetic acid otic - Antifungal therapy: Topical clotrimazole	ND	- Empirical antibacterial therapy: Topical ciprofloxacin (0.3%) -Inflammation therapy: Betamethasone
Outcome		Improved	Improved	No follow-up available	Improved	Improved	Improved

## Data Availability

The genomic data from the present study are submitted under Bioproject number PRJNA1002104.

## Supplementary Materials

The following supporting information can be downloaded at https://www.mdpi.com/article/10.3390/jof9111101/s1, Table S1: Overview of sequence read archive (SRA) numbers of control strains.
