# Supplementary material for: An Autochthonous Susceptible Candida auris Clade I Otomycosis Case in Iran
Source: J Fungi (Basel). 2023 Nov 11;9(11):1101. doi: 10.3390/jof9111101 (PMC10671974; doi:10.3390/jof9111101)
Supplement: Supplementary file 1 [file jof-09-01101-s001.zip › jof-2683795-supplementary.pdf]

**Supplementary Table 1.**

| ID | SRA accession number | Country | Clade |
|---|---|---|---|
| B11203 | SRR1452434 | India | I |
| B11205 | SRR3883436 | India | I |
| B11209 | SRR3883441 | India | I |
| B11213 | SRR3883444 | India | I |
| WM-18.173 | SRR11485330 | India | I |
| B11808 | SRR10461263 | South Korea | II |
| B13463 | SRR10461159 | Canada | II |
| B14308 | SRR10461147 | USA | II |
| B11225 | SRR3883457 | South Africa | III |
| B11229 | SRR3883462 | South Africa | III |
| B11230 | SRR3883463 | South Africa | III |
| B12037 | SRR10461253 | Canada | III |
| B12098 | SRR10461248 | Panama | IV |
| B12177 | SRR10461201 | Venezuela | IV |
| B12336 | SRR7140028 | Colombia | IV |
| B12388 | SRR7909221 | USA | IV |
| MRL40 | SRR18325430 | Iran | V |
| TMML616 | SRR18325431 | Iran | V |
| IFRC2087 | SRR9007776 | Iran | V |
| F0083 | SRR* | Singapore | VI |
| F1580 | SRR* | Singapore | VI |
| F3485 | SRR* | Singapore | VI |
| 102096/2021 | SRR24877249 | Bangladesh | VI |

**SRA: sequence read archive. * submitted**
